# Donepezil Regulates LPS and Aβ-Stimulated Neuroinflammation through MAPK/NLRP3 Inflammasome/STAT3 Signaling

**DOI:** 10.3390/ijms221910637

**Published:** 2021-09-30

**Authors:** Jieun Kim, Hyun-ju Lee, Seon Kyeong Park, Jin-Hee Park, Ha-Ram Jeong, Soojung Lee, Heeyong Lee, Eunyoung Seol, Hyang-Sook Hoe

**Affiliations:** 1 Department of Neural Development and Disease, Korea Brain Research Institute (KBRI), 61, Cheomdan-ro, Dong-gu, Daegu 41062, Korea; jieunkim@kbri.re.kr (J.K.); hjlee@kbri.re.kr (H.-j.L.); skp210316@kbri.re.kr (S.K.P.); mingmeng1005@kbri.re.kr (J.-H.P.); hrj21@kbri.re.kr (H.-R.J.); 2 G2GBIO, Inc., Science Park #411, 1646 Yuseong-daero, Yuseong-gu, Daejeon 34054, Korea; soojung.lee@g2gbio.com (S.L.); heeyong.lee@g2gbio.com (H.L.); eunyoung.seol@g2gbio.com (E.S.); 3 Department of Brain and Cognitive Science, Daegu Gyeongbuk Institute of Science & Technology (DGIST), 333, Techno Jungang-daero, Hyeonpung-eup, Dalseong-gun, Daegu 42988, Korea

**Keywords:** donepezil, rivastigmine, astrocyte, microglia, ROS, NLRP3 inflammasome, STAT3

## Abstract

The acetylcholinesterase inhibitors donepezil and rivastigmine have been used as therapeutic drugs for Alzheimer’s disease (AD), but their effects on LPS- and Aβ-induced neuroinflammatory responses and the underlying molecular pathways have not been studied in detail in vitro and in vivo. In the present study, we found that 10 or 50 μM donepezil significantly decreased the LPS-induced increases in the mRNA levels of a number of proinflammatory cytokines in BV2 microglial cells, whereas 50 μM rivastigmine significantly diminished only LPS-stimulated IL-6 mRNA levels. In subsequent experiments in primary astrocytes, donepezil suppressed only LPS-stimulated iNOS mRNA levels. To identify the molecular mechanisms by which donepezil regulates LPS-induced neuroinflammation, we examined whether donepezil alters LPS-stimulated proinflammatory responses by modulating LPS-induced downstream signaling and the NLRP3 inflammasome. Importantly, we found that donepezil suppressed LPS-induced AKT/MAPK signaling, the NLRP3 inflammasome, and transcription factor NF-kB/STAT3 phosphorylation to reduce neuroinflammatory responses. In LPS-treated wild-type mice, a model of neuroinflammatory disease, donepezil significantly attenuated LPS-induced microglial activation, microglial density/morphology, and proinflammatory cytokine COX-2 and IL-6 levels. In a mouse model of AD (5xFAD mice), donepezil significantly reduced Aβ-induced microglial and astrocytic activation, density, and morphology. Taken together, our findings indicate that donepezil significantly downregulates LPS- and Aβ-evoked neuroinflammatory responses in vitro and in vivo and may be a therapeutic agent for neuroinflammation-associated diseases such as AD.

## 1. Introduction

Neuroinflammation is a critical factor in cognitive dysfunction and neurodegenerative diseases such as Alzheimer’s disease (AD), Parkinson’s disease (PD), Huntington’s disease (HD), and multiple sclerosis (MS) [1,2,3]. Neuroinflammation is initiated when an inflammatory stimulus (Aβ, pathogenic infection or cellular debris) triggers activation of microglia and/or astrocytes in the central nervous system (CNS) [1]. In the brain, abnormal microglial and/or astrocyte activation accelerates the occurrence and development of neuroinflammatory responses [2]. For instance, the neurotoxin lipopolysaccharide (LPS) activates mitogen-activated protein kinase (MAPK) signaling pathways and transcription factor kappa-B (NF-κB)/p-signal transducer and activator of transcription–3 (STAT3) by binding to Toll-like receptor-4 (TLR-4) in in vitro and in vivo models [4,5].

Another inflammatory stimulus and a hallmark of AD, Aβ, leads to neurotoxicity by promoting microglial and astrocyte activation, which in turn triggers the release of proinflammatory cytokines through host cell-derived damage-associated molecular patterns secretion [1]. Moreover, several recent studies have demonstrated that chronic neuroinflammation increases blood–brain barrier (BBB) permeability and accelerates cognitive impairment and brain dysfunction. Therefore, regulating microglial and astrocyte activation as well as neuroinflammation-associated molecular targets may be an effective therapeutic strategy for neurodegenerative diseases.

The acetylcholinesterase (AChE) inhibitors donepezil and rivastigmine are approved for use in AD and other dementias [6]. AChE inhibitors ameliorate AD by inhibiting the breakdown of AChE, a neurotransmitter associated with learning and memory [7]. In addition to their effects on AChE, AChE inhibitors may have other biological functions, such as inhibiting Aβ plaque formation and neuroinflammation in the brain [8,9]. In particular, there is evidence of a correlation between the cholinergic pathway and anti-inflammatory effects [10]. For instance, the α7 nicotinic acetylcholine receptor (α7AChR) has been reported to modulate the inflammatory response by activating the cholinergic anti-inflammatory pathway, and acetylcholine (ACh), a representative neurotransmitter in the cholinergic system, effectively inhibits the activation of macrophages and proinflammatory cytokines [10,11]. These reports suggest that modulating ACh levels via acetylcholinesterase (AChE) inhibitors might also impact neuroinflammatory responses, increasing the therapeutic potential of these drugs. Although the activation of the cholinergic system is expected to affect the regulation of inflammation, whether AChE inhibitors affect LPS- and Aβ-induced neuroinflammatory responses in vivo has not been studied in detail.

The effects of AChE inhibitors also appear to modulate to the NLRP3 (NOD-, LRP-, and pyrin domain-containing protein 3) inflammasome. The NLRP3 inflammasome is formed and activated when endogenous danger signals and environmental irritants are detected by the intracellular sensor NLRP3 [12]. Assembly of the NLRP3 inflammasome leads to the release of the proinflammatory cytokine IL-1β via caspase-1 activation [13]. In BV2 microglial cells, the specific α7nAChR agonist PNU282987 inhibits the LPS-induced increase in NLRP3 inflammasome formation by stimulating α7AChR, a major target of donepezil [14]. In addition, treatment with the cholinergic receptor agonist GTS-21 or vagus nerve stimulation significantly inhibits NLRP3 inflammasome activation in wild-type mice, whereas genetic deletion of α7nAChR in α7nAChR KO mice enhances NLRP3 inflammasome formation [15]. Vagus nerve stimulation releases ACh, and in cultured human macrophages, ACh release leads to dephosphorylation of NF-kB or STAT3 and suppression of IL-1β and TNF-α cytokine release [16,17,18]. These observations indicate that ACh levels in the synaptic cleft are responsible for modulating neuroinflammatory pathways related to NLRP3 inflammasome formation, such as NF-kB/STAT3, but the effects of AChE inhibitors on other MAPK-associated NLRP3 inflammasome pathways have not been established in detail.

In the present study, we evaluated the effects of the AChE inhibitors donepezil and rivastigmine on LPS- or Aβ-mediated neuroinflammatory responses in vitro and in vivo. Both donepezil and rivastigmine effectively attenuated LPS-stimulated proinflammatory cytokines in BV2 microglial cells. Examination of the molecular mechanism by which donepezil affected neuroinflammation demonstrated that donepezil impacted LPS-stimulated AKT/MAPK signaling, NLRP3 inflammasome formation, and downstream NF-kB/STAT3 signaling in BV2 microglial cells. In wild-type mice, donepezil modulated the induction of microgliosis and COX-2 and IL-6 expression by LPS. Furthermore, in 5xFAD mice, donepezil treatment significantly reduced Aβ-evoked activation of microglia and astrocytes. Overall, these data suggest that donepezil may be a potential therapeutic drug for neuroinflammation-associated diseases.

## 2. Results

### 2.1. Donepezil Reduces LPS-Mediated Proinflammatory Cytokine mRNA Levels in BV2 Microglial Cells

**Figure 1:**
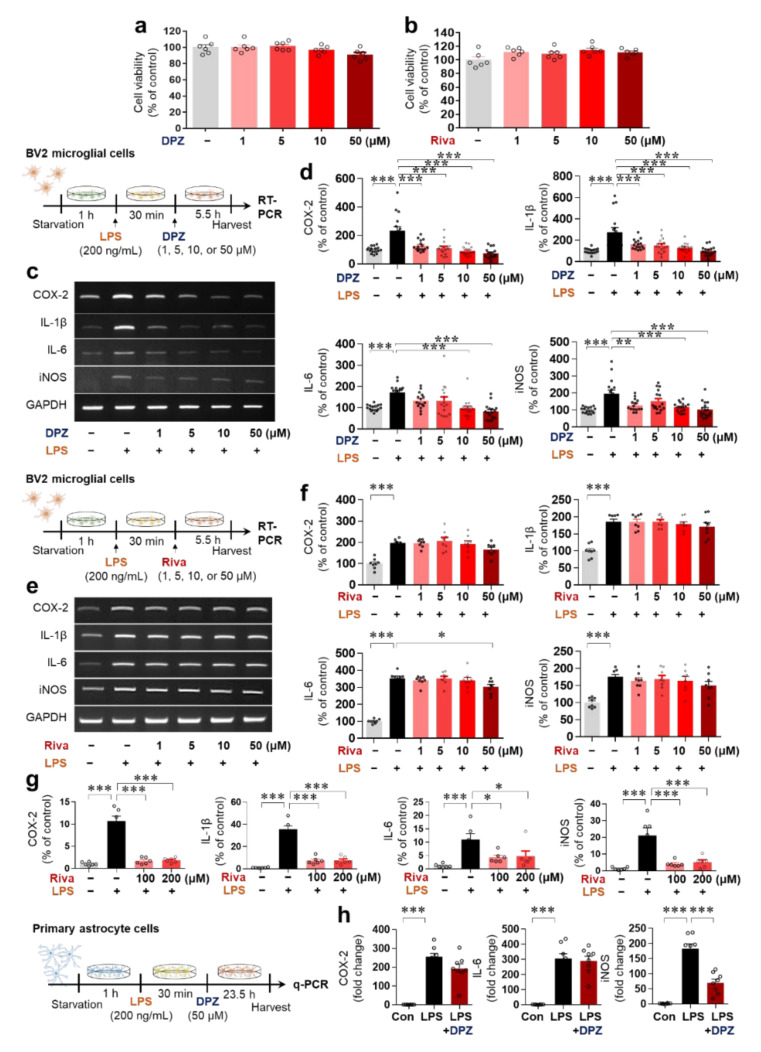
Donepezil ameliorates the induction of proinflammatory cytokines by LPS in BV2 microglial cells and primary astrocytes. (**a**,**b**) The cytotoxicity of donepezil (**a**) and rivastigmine (**b**) was evaluated by the MTT assay in cells treated with a range of concentrations (1, 5, 10, and 50 μM) or vehicle (1% DMSO) for 24 h (*n* = 6/group). (**c**,**d**) RT-PCR analysis of proinflammatory cytokine levels in BV2 microglial cells pretreated with LPS or PBS and treated with donepezil or vehicle as shown (*n* = 16/group). (**e**,**f**) RT-PCR analysis of proinflammatory cytokine levels in BV2 microglial cells pretreated with LPS or PBS and treated with rivastigmine or vehicle (1% DMSO) as shown (*n* = 8/group). (**g**) q-PCR analysis of proinflammatory cytokine levels in BV2 microglial cells pretreated with LPS or PBS and treated with 100 or 200 μM rivastigmine or vehicle (1% DMSO) as in (**e**,**f**) (*n* = 6/group). (**h**) q-PCR analysis of proinflammatory cytokine levels in primary astrocytes pretreated with LPS or PBS and treated with donepezil or vehicle (1% DMSO) as shown (*n* = 8/group). * *p*< 0.05, ** *p* < 0.01, *** *p* < 0.001.

Before examining the effects of donepezil and rivastigmine on LPS-mediated proinflammatory cytokine levels, we first assessed the cytotoxicity of donepezil and rivastigmine at concentrations of 1–50 μM in BV2 microglial cells using the MTT assay. Neither donepezil nor rivastigmine exhibited cytotoxicity at doses of up to 50 μM (Figure 1a,b).

Next, we assessed the effects of these doses of donepezil and rivastigmine (1–50 μM) on LPS-mediated proinflammatory cytokine levels. Analysis of proinflammatory cytokines by RT-PCR showed that donepezil significantly reduced LPS-mediated COX-2, IL-1β, IL-6 and iNOS mRNA levels in BV2 microglial cells (Figure 1c,d), whereas 50 μM rivastigmine significantly decreased only LPS-mediated IL-6 mRNA levels (Figure 1e,f). Treatment with higher doses of rivastigmine (100 or 200 μM) significantly suppressed LPS-induced COX-2, IL-1β, IL-6, and iNOS mRNA levels in BV2 microglial cells (Figure 1g). Overall, donepezil reduced the induction of proinflammatory cytokine levels by LPS more effectively than rivastigmine in BV2 microglial cells.

### 2.2. Donepezil Suppresses Only LPS-Induced iNOS mRNA Levels in Primary Astrocytes

To further assess the effects of donepezil on LPS-induced proinflammatory cytokine levels in vitro, primary astrocyte cells were pretreated with 200 ng/mL LPS or PBS for 30 min and treated with 50 μM donepezil or vehicle (1% DMSO) for 23.5 h. q-PCR analysis of proinflammatory cytokine levels indicated that donepezil significantly reduced the LPS-evoked mRNA levels of iNOS, but not other proinflammatory cytokines (Figure 1h). These results suggest that donepezil downregulates LPS-induced neuroinflammatory responses more effectively in microglia than in astrocytes in vitro.

### 2.3. Donepezil and Rivastigmine Independently Modulate LPS-Induced Intracellular ROS Production and Mitochondrial Dysfunction

**Figure 2:**
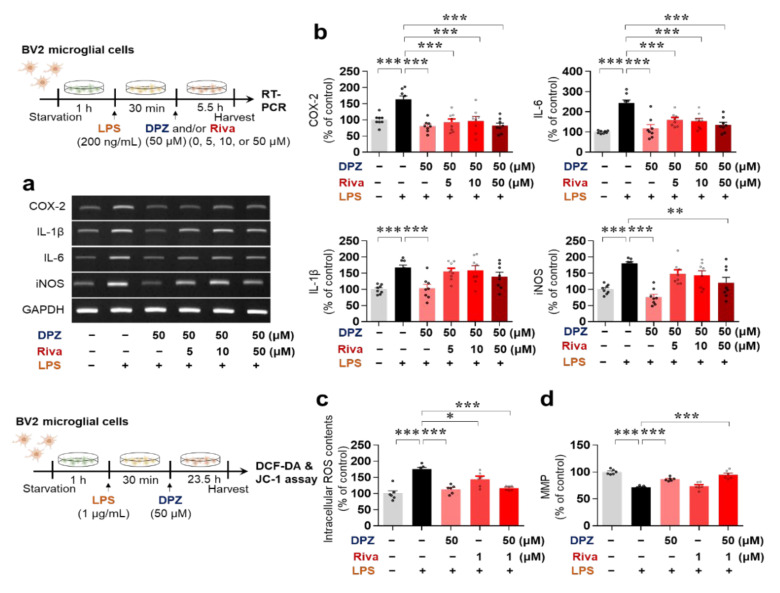
Donepezil affects LPS-stimulated ROS production and mitochondrial function in BV2 microglial cells. (**a**,**b**) RT-PCR analysis of proinflammatory cytokine levels in BV2 microglial cells pretreated with LPS or PBS and co-treated with 50 μM donepezil and a range of rivastigmine concentrations as shown (*n* = 6/group). (**c**,**d**) Intracellular ROS content and mitochondrial membrane potential damage, as evaluated by DCF-DA and the JC-1 assay, respectively, in BV2 microglial cells pretreated with LPS or PBS and treated with donepezil and/or rivastigmine as shown (*n* = 6/group). * *p* < 0.05, ** *p* < 0.01, *** *p* < 0.001.

The potential synergism between the effects of donepezil and rivastigmine on LPS-evoked neuroinflammatory responses was evaluated in BV2 microglial cells pretreated with 1 μg/mL LPS or PBS for 30 min and co-treated with 50 μM donepezil and 0, 5, 10, or 50 μM rivastigmine or vehicle for 23.5 h. Confirming the results in Figure 1, donepezil alone significantly decreased the induction of proinflammatory cytokine mRNA levels by LPS (Figure 2a,b). However, co-treatment with donepezil and rivastigmine did not produce synergistic effects on LPS-mediated proinflammatory cytokine levels compared with donepezil treatment alone (Figure 2a,b).

Mitochondrial dysfunction promotes neuroinflammation, and conversely, the neuroinflammatory response causes mitochondrial dysfunction, leading to increased intracellular ROS content due to an imbalance between ROS generation and removal [19,20]. We therefore examined whether donepezil and rivastigmine independently or synergistically regulate intracellular ROS production and mitochondrial function. BV2 microglial cells were pretreated with 1 μg/mL LPS or PBS for 30 min and treated with 50 μM donepezil, 1 μM rivastigmine, 50 μM donepezil plus 1 μM rivastigmine, or vehicle for 23.5 h. Both donepezil or rivastigmine significantly reduced LPS-induced intracellular ROS production in BV2 microglial cells (Figure 2c), but donepezil protected against LPS-mediated mitochondrial membrane potential damage more effectively than rivastigmine (Figure 2d). No synergistic effects of co-treatment with donepezil and rivastigmine on LPS-mediated ROS levels (Figure 2c) or mitochondrial function (Figure 2d) were observed. These data indicate that donepezil regulates LPS-stimulated ROS production and mitochondrial dysfunction in BV2 microglial cells.

### 2.4. Donepezil Reduces LPS-Induced AKT/MAPK Signaling and Nuclear p-NF-κB/p-STAT3 Levels in BV2 Microglial Cells

**Figure 3:**
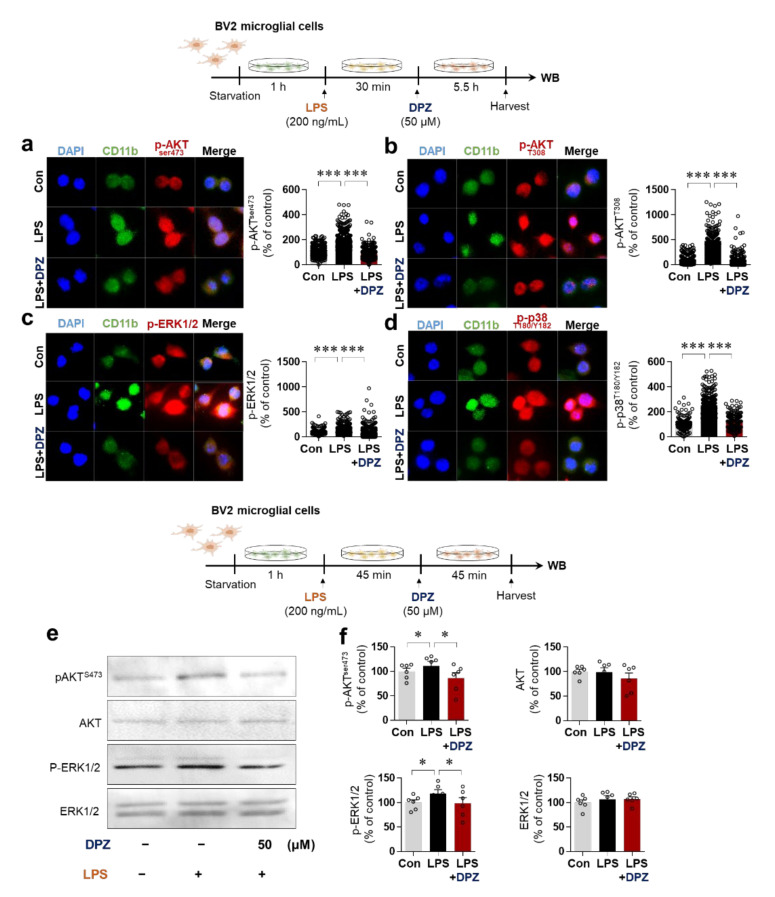
Donepezil affects LPS-mediated AKT/MAPK signaling in BV2 microglial cells. (**a**,**b**) p-AKT^ser473^, p-AKT^T308^, and CD11b expression in cells pretreated with LPS (200 ng/mL) or PBS and treated with donepezil (50 μM) or vehicle (1% DMSO) as shown at the top of the figure (p-AKT^ser473^; Con: *n* = 434, LPS: *n* = 478, LPS + Donepezil: *n* = 400; p-AKT^T308:^ Con: *n* = 231PS: *n* = 231, LPS + Donepezil: *n* = 206). (**c**,**d**) p-ERK, p-P38^T180/Y182^, and CD11b expression in cells treated as in (**a**,**b**) (p-ERK1/2(p44/42): Con: *n* = 189, LPS: *n* = 195, LPS + Donepezil: *n* = 233; p-P38^T180/Y182^: Con: *n* = 191, LPS: *n* = 231, LPS + Donepezil: *n* = 217). (**e**,**f**) Western blot analysis of p-AKT, AKT, p-ERK, and ERK levels in cells pretreated with LPS (200 ng/mL) or PBS and treated with donepezil (50 μM) or vehicle as shown in the middle of the figure (*n* = 6/group). * *p* < 0.05, *** *p* < 0.001.

Glial cell activation is associated with the AKT and MAPK signaling pathways induced by LPS, including JNK, P38 and ERK [21]. To evaluate the impact of donepezil on LPS-induced AKT/MAPK signaling, BV2 microglial cells were pretreated with LPS or PBS and treated with donepezil (50 μM) or vehicle (1% DMSO) as shown in the top half of Figure 3. ICC analysis using anti-p-AKT^ser473^, anti-p-AKT^T308^, anti-p-ERK (p44/p42) or anti-p-P38^T180/Y18^ with anti-CD11b revealed that donepezil significantly reduced LPS-stimulated p-AKT^ser473^, p-AKT^T308^, p-ERK and p-P38 ^T180/Y18^ levels in BV2 microglial cells (Figure 3a–d). Subsequent Western blot analysis of BV2 microglial cells pretreated with LPS or PBS and treated with donepezil or vehicle as shown in the bottom half of Figure 3 confirmed the significant reductions in LPS-mediated p-AKT^ser473^ and p-ERK levels (Figure 3e,f). These data suggest that donepezil alters LPS-stimulated neuroinflammatory responses by modulating AKT/MAPK signaling.

**Figure 4:**
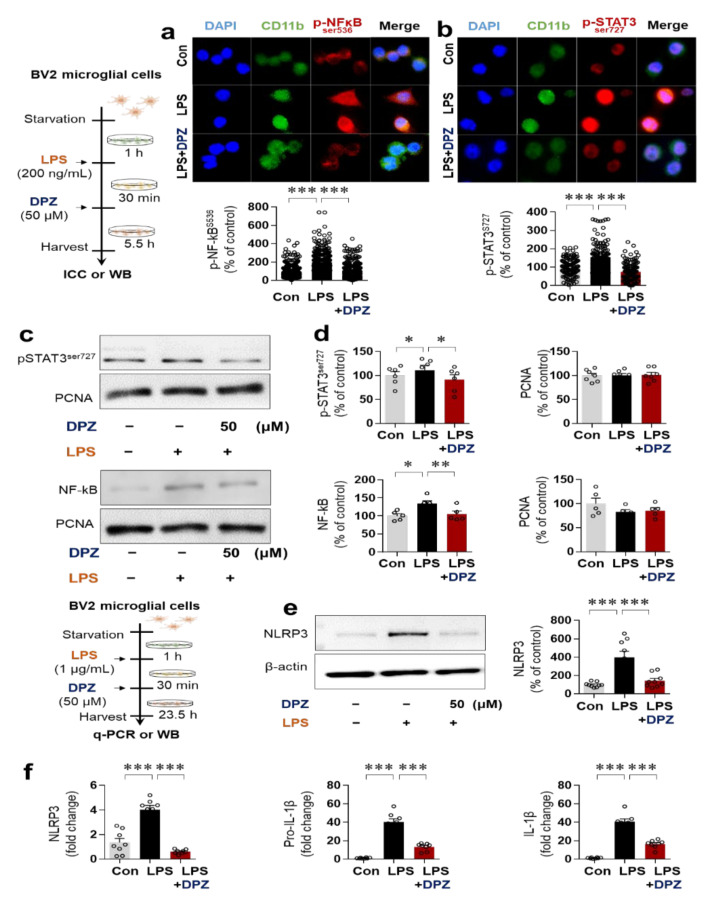
Donepezil regulates LPS-induced NF-κB/STAT3 phosphorylation and NLRP3 inflammasome formation in BV2 microglial cells. (**a**,**b**) p-NFκB^Ser536^, p-STAT3^Ser727^, and CD11b expression, as determined by ICC analysis (p-NF-kB^ser536^: Con: *n* = 353, LPS: *n* = 331, LPS + Donepezil: *n* = 339; p-STAT3^ser727^: Con: *n* = 133, LPS: *n* = 148, LPS + Donepezil: *n* = 138). (**c**,**d**) Nuclear fractionation analysis of p-STAT3, NF-kB, and PCNA in cells pretreated with 200 ng/mL LPS or PBS for 30 min and treated with 50 μM donepezil or vehicle (1% DMSO) for 5.5 h, as determined by Western blot (*n* = 6–7/group). I NLRP3 protein expression levels in cells pretreated with 1 μg/mL LPS or PBS for 30 min and treated with 50 μM donepezil or vehicle (1% DMSO) for 23.5 h, as determined by Western blot (*n* = 9/group). (**f**) NLRP3 inflammasome formation in cells pretreated with 1 μg/mL LPS or PBS for 30 min and treated with 50 μM donepezil or vehicle (1% DMSO) for 23.5 h, as determined by q-PCR (*n* = 8/group). * *p* < 0.05, ** *p* < 0.01, *** *p* < 0.001.

Next, we investigated the transcription factor requirements for the effects of donepezil on LPS-induced neuroinflammation in ICC assays using anti-p-NF-κB^Ser536^ or anti-p-STAT3^Ser727^. In BV2 microglial cells, donepezil treatment effectively suppressed LPS-induced nuclear p-NF-kB^Ser536^ levels, consistent with a previous report [22], and significantly decreased LPS-evoked nuclear p-STAT3(ser727) levels (Figure 4a,b). Nuclear fractionation and subsequent Western blotting with anti-p-NF-κB^Ser536^, anti-p-STAT3^Ser727^, or anti-PCNA (a nucleus marker) confirmed that donepezil treatment decreased LPS-mediated p-NF-kB ^Ser536^ and p-STAT3 ^Ser727^ levels in the nucleus in BV2 microglial cells (Figure 4c,d). Our findings suggest that donepezil reduces LPS-evoked neuroinflammatory responses by altering AKT/MAPK-linked signaling and nuclear STAT3/NF-kB phosphorylation in BV2 microglial cells.

### 2.5. Donepezil Ameliorates LPS-Induced NLRP3 Inflammasome Formation in BV2 Microglial Cells

Since donepezil reduced LPS-mediated IL-1β mRNA levels in BV2 microglial cells, we investigated whether donepezil affects LPS-induced neuroinflammatory responses via NLRP3 inflammasome formation. BV2 microglial cells were pretreated with 1 μg/mL LPS or PBS for 30 min and treated with 50 μM donepezil or vehicle (1% DMSO) for 23.5 h. Subsequent Western blotting with anti-NLRP3 demonstrated that donepezil significantly reduced LPS-induced NLRP3 levels (Figure 4e). In addition, q-PCR analysis showed that donepezil significantly suppressed LPS-activated NLRP3, pro-IL-1β, and IL-1β mRNA levels (Figure 4f). These data indicate that donepezil regulates LPS-stimulated neuroinflammation by inhibiting NLRP3 inflammasome formation in BV2 microglial cells.

### 2.6. Donepezil Downregulates LPS-Mediated Microglial Density, Morphology and Activation in Wild-Type Mice

**Figure 5:**
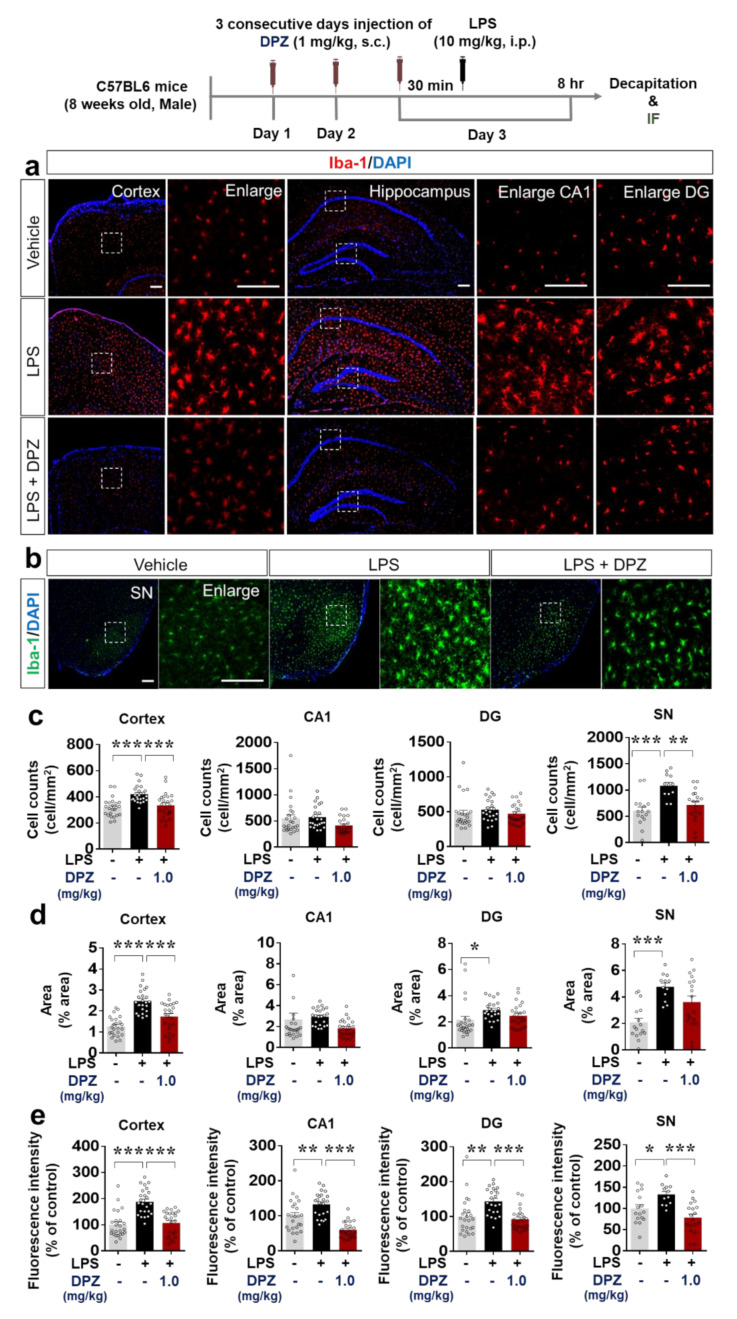
Donepezil suppresses LPS-evoked microgliosis and hypertrophy in wild-type mice. (**a**,**b**) Iba-1 expression in brain sections from mice treated as shown, as determined by immunostaining with anti-Iba-1. (**c**–**e**) Quantification of the results in (**a**,**b**) (*n* = 12–24 brain slices from 4 mice/group). * *p* < 0.05, ** *p* < 0.01, *** *p* < 0.001.

To evaluate the inhibition of LPS-mediated microglial activation by donepezil in vivo, wild-type mice were injected once daily with 1 mg/kg donepezil (s.c.) or vehicle (saline) for 3 days. Thirty minutes after the last donepezil injection, 10 mg/kg LPS (i.p.) or PBS was injected. Eight hours later, the mice were sacrificed, and immunohistochemistry was performed with anti-Iba-1 to determine the number of Iba-1-positive cells, the percentage stained area and fluorescence intensity of Iba-1 in the cortex, hippocampus (DG and CA1), and substantia nigra (SN). Donepezil treatment significantly decreased the number of Iba-1-positive cells induced by LPS in the cortex and SN, but not the hippocampus (Figure 5a–c). Donepezil significantly reduced the LPS-mediated percentage stained area of Iba-1 in the cortex only (Figure 5a–d). Donepezil also significantly reduced the LPS-mediated fluorescence intensity of Iba-1 in the cortex, hippocampus (DG and CA1), and SN (Figure 5a,b,e). These data suggest that donepezil modulates LPS-stimulated microgliosis by attenuating changes in the morphology and activation of microglia.

### 2.7. Donepezil Decreases the Induction of COX-2 and IL-6 Levels by LPS in Wild-Type Mice

**Figure 6:**
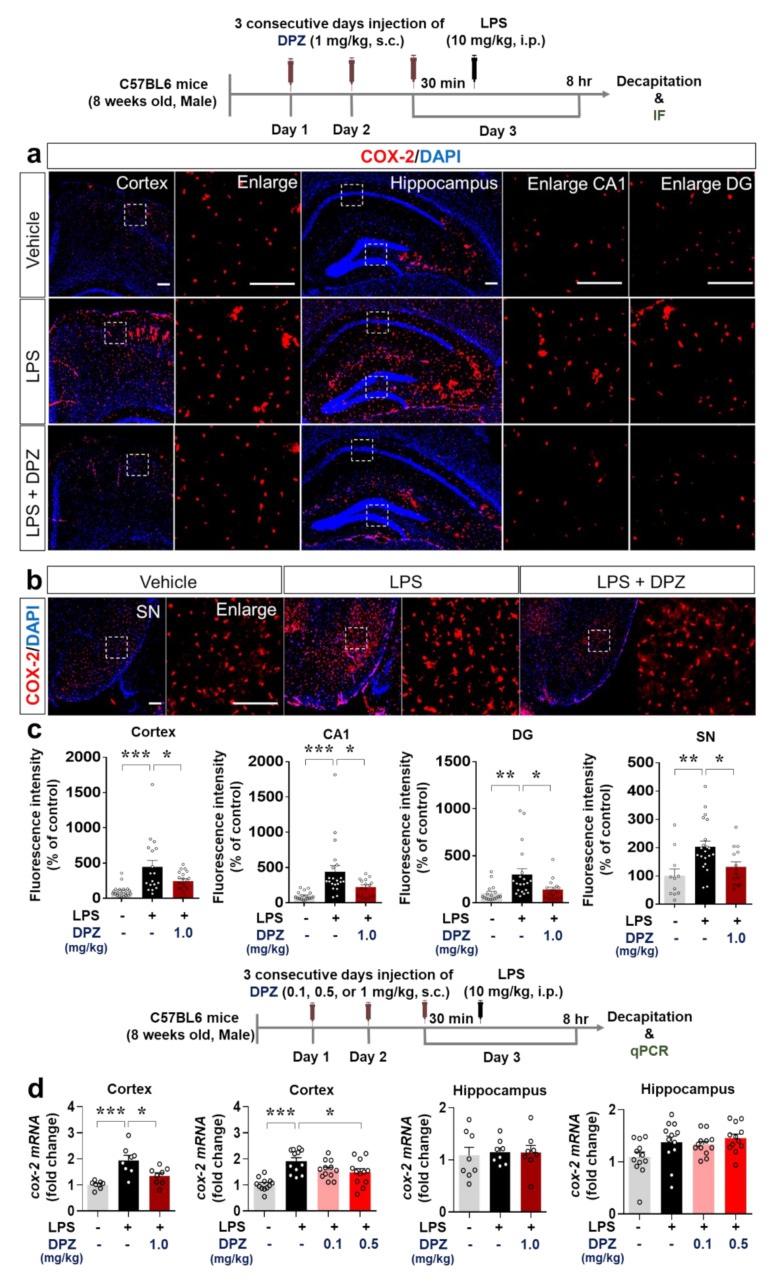
Donepezil reduces the stimulation of COX-2 levels by LPS in wild-type mice. (**a**,**b**) Immunostaining of brain slices from wild-type mice treated as shown with anti-COX-2. (**c**) Quantification of the results in (**a**,**b**) (*n* = 11–21 brain slices from 4 mice/group). (**d**) q-PCR analysis of COX-2 mRNA levels in brain slices from wild-type mice treated as shown (*n* = 8–12/group). * *p* < 0.05, ** *p* < 0.01, *** *p* < 0.001.

**Figure 7:**
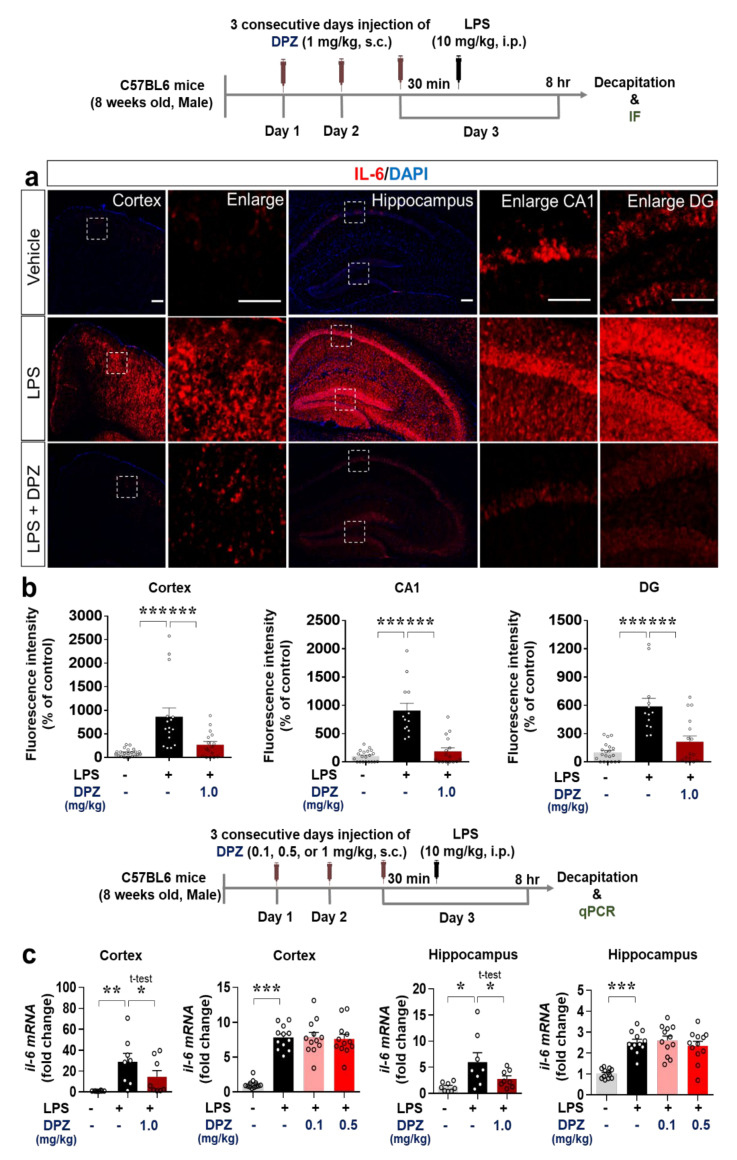
Donepezil decreases the stimulation of IL-6 levels by LPS in wild-type mice. (**a**,**b**) Immunostaining of brain slices from wild-type mice treated as shown with anti-IL-6. (**b**) Quantification of the results in (**a**) (*n* = 13–22 brain slices from 4 mice/group). (**c**) q-PCR analysis of IL-6 mRNA levels in brain slices from wild-type mice treated as shown (*n* = 8–12/group) * *p* < 0.05, ** *p* < 0.01, *** *p* < 0.001.

Since donepezil affected LPS-induced microglial activation and morphology, we examined the ability of donepezil to modulate LPS-evoked proinflammatory cytokine levels in vivo. Wild-type mice were treated with 1 mg/kg donepezil and LPS as described in Section 2.6, and immunohistochemistry was performed with antibodies against the proinflammatory cytokines COX-2 and IL-6. Donepezil (1 mg/kg, s.c.) significantly decreased the induction by LPS of COX-2 in the cortex, hippocampus, and SN (Figure 6a–c) and IL-6 in the cortex and hippocampus (Figure 7a,b).

To obtain confirmation of the RT-PCR results, we assessed COX-2 and IL-6 mRNA levels by q-PCR. Cortical but not hippocampal COX-2 mRNA levels were significantly reduced in wild-type mice injected with 0.5 or 1 mg/kg donepezil followed by 10 mg/kg LPS (i.p.) or PBS (Figure 6d). No changes in COX-2 mRNA levels were observed in either the cortex or hippocampus when a donepezil dose of 0.1 mg/kg was used (Figure 6d). LPS-evoked IL-6 mRNA levels in the cortex and hippocampus were downregulated by 1 mg/kg donepezil (Figure 7c), whereas doses of 0.1 and 0.5 mg/kg had no effect (Figure 7c). These data demonstrate that 1 mg/kg donepezil is an effective dose for decreasing the levels of COX-2 and IL-6 proinflammatory cytokines induced by LPS in wild-type mice.

### 2.8. Donepezil Reduces Aβ-Mediated Neuroinflammation in a Mouse Model of AD

**Figure 8:**
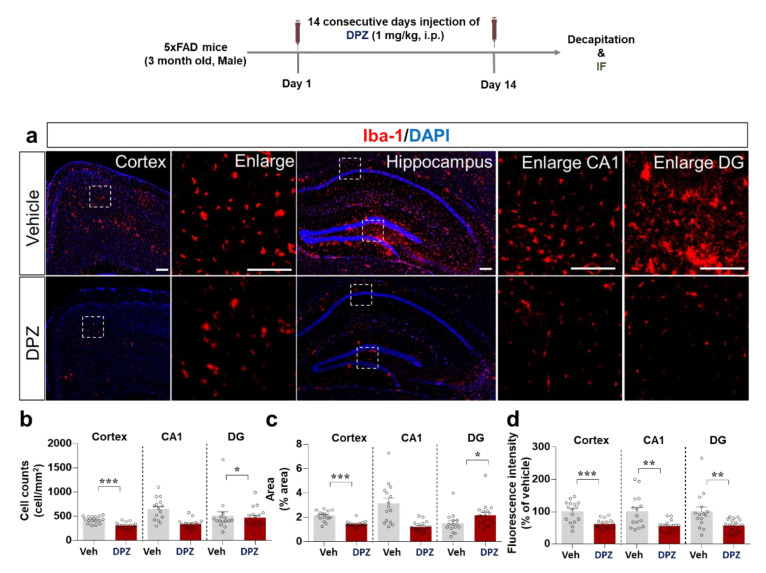
Donepezil suppresses Aβ-mediated microgliosis and hypertrophy in a mouse model of AD. (**a**) Immunostaining of Iba-1 in brain sections from 5xFAD mice treated as shown with anti-Iba-1. (**b**–**d**) Quantification of the results in (**a**) (*n* = 15–18 brain slices from 4 mice/group). * *p* < 0.05, ** *p* < 0.01, *** *p* < 0.001.

Aβ is a critical pathological factor in AD and it promotes neuroinflammation in the brain [23]. Therefore, we evaluated the effects of donepezil on Aβ-stimulated neuroinflammation in 5xFAD mice, a model of AD. The mice were injected with 1 mg/kg donepezil (i.p.) or vehicle (10% DMSO) daily for 2 weeks. Immunostaining of brain sections with anti-Iba-1 showed that donepezil treatment significantly suppressed the percentage anti-Iba-1-stained area and number of Iba-1-positive cells in the cortex and hippocampal DG region but not the hippocampal CA1 region (Figure 8a–c). In addition, donepezil significantly downregulated cortical and hippocampal Iba-1 intensity (Figure 8a,d).

**Figure 9:**
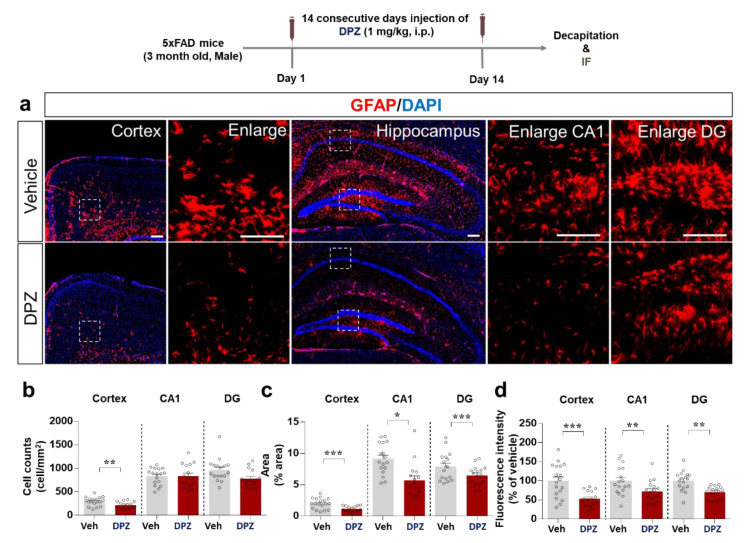
Donepezil attenuates Aβ-mediated astrogliosis and hypertrophy in 5xFAD mice. (**a**) Immunostaining of brain sections from 5xFAD mice treated as shown with anti-GFAP. (**b**–**d**) Quantification of the results in (**a**) (*n* = 16–18 brain slices from 4 mice/group). * *p* < 0.05, ** *p* < 0.01, *** *p* < 0.001.

The effects of donepezil on Aβ-stimulated astrogliosis were examined by immunostaining of brain sections from 5xFAD mice treated as described above with anti-GFAP. Interestingly, donepezil significantly reduced the number of GFAP-positive cells in the cortex but not the hippocampus (CA1 and DG regions) (Figure 9a,b). Moreover, GFAP intensity and the percentage anti-GFAP-stained area in the cortex and hippocampus were significantly reduced by donepezil treatment (Figure 9c,d). These data indicate that donepezil alters Aβ-stimulated microglial and astrocyte activation as well as morphology in this model of AD.

## 3. Discussion

Microglial and astrocyte activation contributes to the pathogenesis of cognitive dysfunction and neurodegenerative disease [24]. In the present study, donepezil effectively modulated neuroinflammatory responses induced by LPS or Aβ in vitro and in vivo. Specifically, donepezil treatment reduced the effects of LPS on proinflammatory cytokine levels, intracellular ROS production, and mitochondrial function in BV2.

Microglial cells had a smaller effect on LPS-induced proinflammatory cytokine levels in primary astrocytes. Donepezil also downregulated LPS-induced AKT/MAPK signaling, NLRP inflammasome activation, and nuclear NF-kB/STAT3 phosphorylation in BV2 microglial cells. In wild-type mice, donepezil significantly downregulated LPS-evoked microgliosis and proinflammatory cytokine levels in the brain. Importantly, micro- and astrogliosis were significantly suppressed in donepezil-treated 5xFAD mice (a mouse model of AD). Overall, our findings suggest that donepezil alters LPS- and Aβ-stimulated neuroinflammation in the brain.

The first generation of FDA-approved drugs for the treatment of AD pathology focused on directly inhibiting Aβ production or α-secretase activity. However, recent studies have revealed that neuroinflammation is a critical target for treating AD pathology [25]. The process of neuroinflammation is related to the onset of several neurodegenerative disorders and an important contributor to AD pathogenesis and progression as well as reduced cognitive function [26]. Therefore, novel AD treatments directed at neuroinflammation and its related mechanisms are being developed. In line with these efforts, in this study we verified the effects of the AChE inhibitor donepezil on neuroinflammation using two different neuroinflammation-associated disease models (i.e., LPS-induced or a mouse model of AD).

Anti-inflammatory effects of AChE inhibitors on various stimulus-induced inflammatory responses have been observed in vitro and in vivo. For example, AChE inhibition alters the inflammatory response by modulating ACh levels and the activation of α7 nicotinic AChRs. In RAW 264.7 murine macrophage cells, AChE inhibitors, including donepezil, significantly reduce LPS-induced upregulation of the proinflammatory cytokines TNF-α, IL-1β, IL-2, IL-6, and IL-18 and the NF-κB signaling pathway [27]. Donepezil was previously shown to attenuate iNOS, IL-1β, and TNF-α levels in LPS-stimulated BV2 microglial and primary microglial cells [28] and to modulate microglial activation by regulating mTOR signaling in a high-fat diet-induced neuroinflammation model [29]. In rat chemobrain induced by the anticancer drug doxorubicin, donepezil significantly suppresses neuroinflammatory responses (i.e., TNF- α, IL-6, Iba-1, GFAP), the expression of oxidative stress markers (i.e., MDA, GPx4, SOD2), and mitochondrial dysfunction (i.e., ROS, mitochondrial membrane potential) [30]. In addition, post-treatment with donepezil inhibits Aβ-mediated upregulation of proinflammatory mediators (i.e., NO, iNOS, IL-1β, TNF-α, COX-2, Mac-1, or GFAP) and p38/p65 signaling pathway in BV2 microglial cells, rat primary microglial cells, and mouse brain [11]. In THP-1 macrophages (an in vitro model of vascular dementia), donepezil but not rivastigmine significantly inhibits COX-1 mRNA levels [31], as does co-treatment with 100 ng/mL donepezil and 25 ng/mL rivastigmine, although co-treatment at lower doses has no effect [31]. Interestingly, galantamine, tacrine, and physostigmine, which are also AChE inhibitors, do not affect LPS-stimulated inflammatory responses in RAW 264.7 murine macrophage cells [27]. In the present study, donepezil significantly suppressed the induction of the mRNA expression of COX-2, IL-6, IL-1β, and iNOS in BV2 microglial cells (Figure 1). Compared with donepezil, rivastigmine had smaller effects on LPS-mediated proinflammatory cytokine levels in this cell line, indicating that donepezil alters LPS-induced microglial neuroinflammatory responses more effectively than rivastigmine (Figure 1). In primary astrocytes, only LPS-stimulated iNOS mRNA levels were significantly reduced by donepezil treatment; the mRNA levels of other proinflammatory cytokines were not affected (Figure 1), suggesting that donepezil modulates LPS-evoked neuroinflammation more potently in microglia than in primary astrocytes.

Although donepezil is approved by the FDA to treat AD, its effect on AChE is short-lived, and tolerance may occur in humans [32]. Rivastigmine was recently approved for the treatment of AD based on its prolonged inhibition of AChE for 10 h in the CNS compared with the peripheral system [3,33]. Several recent studies have sought to overcome the short effectiveness and tolerance of donepezil by combining donepezil with memantine (an NMDA agonist) or rivastigmine. For instance, in THP-1 macrophages, co-treatment with donepezil (100 ng/mL) and rivastigmine (25 ng/mL) downregulates gene and protein levels of members of the COX family (e.g., COX-1 and COX-2) [31]. However, the effects of co-treatment with donepezil and rivastigmine on LPS-induced IL-1β, IL-6, and iNOS levels have not been fully investigated. Based on previous findings, we hypothesized that donepezil and rivastigmine co-treatment might alter the levels of other LPS-induced proinflammatory cytokines. In fact, co-treatment with donepezil (50 μM) and rivastigmine (50 μM) significantly reduced COX-2, IL-6 and iNOS mRNA levels induced by LPS (Figure 2). Unexpectedly, IL-1β and iNOS mRNA levels exhibited a tendency to increase in cells co-treated with donepezil (50 μM) and rivastigmine (5, 10 μM) compared with donepezil alone (Figure 2), suggesting that donepezil and rivastigmine do not synergistically affect LPS-evoked proinflammatory cytokine levels.

Why does co-treatment with donepezil (50 μM) and rivastigmine (5, 10 μM) increase LPS-evoked IL-1β and iNOS mRNA levels compared with donepezil treatment alone? There are a few possible explanations for this finding. First, donepezil and rivastigmine may differentially regulate LPS-stimulated downstream signaling pathways, AKT/MAPK signaling and transcription factors (i.e., NF-kB, STAT3) to alter LPS-stimulated IL-1β and iNOS mRNA levels. Several studies have reported that AKT/MAPK signaling and the transcription factors STAT3 and NF-kB modulate LPS-induced proinflammatory cytokine levels. For example, AKT phosphorylation increases LPS-induced IL-1β mRNA levels by activating p-STAT3 in BV2 microglial cells [34]. In LPS-treated murine macrophage cells, enhanced phosphorylation of STAT3 at Y705 is associated with increased IL-1β mRNA levels [35], and phosphorylation of STAT3 at S727 is required for LPS-evoked IL-1β mRNA and protein expression in mouse primary macrophages [36]. In addition, NF-kB is a key modulator of LPS-stimulated IL-1β mRNA and protein expression in mouse primary neutrophils [37]. Here, we observed that donepezil altered LPS-mediated AKT signaling and nuclear NF-kB and STAT3 phosphorylation, and thus we suggest that donepezil and rivastigmine compete to alter LPS-mediated AKT signaling and/or nuclear STAT3 and NF-kB phosphorylation when administered together compared with administration of either alone, resulting in increased LPS-stimulation of IL-1β mRNA levels. The effects of rivastigmine on LPS-evoked AKT/MAPK signaling and NF-kB/STAT3 remain to be elucidated. Future work will include an investigation of whether rivastigmine selectively regulates LPS-induced AKT/MAPK signaling and NF-kB/STAT3 to modulate LPS-mediated proinflammatory cytokine levels. In addition, we will investigate whether donepezil and rivastigmine co-treatment differentially regulates LPS-induced AKT/MAPK signaling and transcription factors compared with administration of either AChE inhibitor alone.

A second possibility is that donepezil and rivastigmine compete to regulate AChE activity when administered together. Modulation of AChE activity is required to suppress IL-1β levels in the mouse peripheral system (i.e., blood) and brain [38]. The differential alteration of LPS-mediated proinflammatory cytokine mRNA levels by donepezil and rivastigmine co-treatment compared with administration of either drug alone may reflect a greater impact of co-treatment on AChE activity. Therefore, future work will identify the optimal dose and ratio for co-treatment with donepezil and rivastigmine to reduce the LPS-induced increment of proinflammatory cytokine levels.

The duration of treatment of primary astrocytes with donepezil was 6 or 24 h, and it is possible that donepezil differentially affects LPS-induced astrocytic neuroinflammatory responses in a time-dependent manner. Future work will examine whether donepezil selectively and/or differentially regulates LPS-stimulated astrocytic proinflammatory cytokine levels in a time-dependent manner. Mitochondrial dysfunction leads to neuroinflammation; conversely, the neuroinflammatory response can cause mitochondrial dysfunction [39]. This interdependence ultimately contributes to the progression of neurodegenerative diseases. Interestingly, several recent studies have found that LPS treatment promotes mitochondrial impairment, resulting in increased intracellular ROS production due to an imbalance of ROS generation and removal (redox) [40,41]. As a result, modulators of oxidative stress and mitochondrial function are attracting attention as novel therapeutic targets in neurodegenerative diseases [42,43]. In this study, treatment with donepezil or rivastigmine alone significantly attenuated LPS-stimulated intracellular ROS levels in BV2 microglial cells (Figure 2c,d). However, no synergistic effects of co-treatment with donepezil and rivastigmine on LPS-stimulated intracellular ROS production were observed (Figure 2c,d). Donepezil and rivastigmine have been shown to reduce oxidative stress in AD patients [40], but evidence suggests that these two drugs regulate oxidative stress via different mechanisms. Donepezil modulates the glutathione-associated signaling pathway to alter oxidative stress, whereas rivastigmine inhibits glycation signaling by reducing the level of advanced glycation end-products (AGEs), a marker of biomolecular peroxidation in AD patients [40]. Interestingly, donepezil ameliorates mitochondrial swelling and ATP levels in Aβ-treated SD rats [44]. We found that donepezil, but not rivastigmine, ameliorated LPS-mediated mitochondrial dysfunction in BV2 microglial cells (Figure 2c,d). In combination with the literature, our findings suggest that donepezil and/or rivastigmine modify LPS- and Aβ-induced intracellular ROS levels, oxidative stress, and mitochondrial dysfunction in vitro and in vivo. The modulation of LPS-induced proinflammatory cytokine levels, ROS production, and mitochondrial dysfunction (Figure 1 and Figure 2) by donepezil raises the question of how donepezil alters LPS-induced neuroinflammation. Donepezil was previously shown to inhibit LPS-stimulated neuroinflammatory responses through binding to an unknown receptor other than TLR-4 in human-derived immune cell models [45]. In human umbilical vein endothelial cells, donepezil decreases endothelial permeability by modulating PI3K/AKT/NF-kB signaling [46]. In the present study, donepezil suppressed LPS-mediated AKT/ERK/P38 signaling (Figure 3) and LPS-induced nuclear p-STAT3/p-NF-kB levels in BV2 microglial cells (Figure 4). In addition, donepezil, which indirectly activates α7nAChR, significantly reduced LPS-mediated NLRP3 inflammasome activation, a key factor in neuroinflammation and neurodegenerative disease, in this cell line (Figure 4e,f). When combined with previous findings, our observations suggest that donepezil affects LPS-evoked AKT/MAPK signaling, NLRP3 inflammasome formation, and NF-kB/STAT3 signaling to alter neuroinflammatory responses. However, other possibilities exist. Interestingly, treatment of RAW 264.7 macrophage cells with LPS leads to α7nAChR inhibition, and donepezil suppresses LPS-stimulated proinflammatory cytokine TNF-α levels and NF-κB signaling in this cell line [47]. In addition, α7nAChR has been speculated to function upstream of NLRP3 inflammasome signaling in microglia [48]. Therefore, it is possible that donepezil rescues LPS-induced α7nAChR inactivation by increasing ACh levels to modulate neuroinflammatory responses in microglial cells. We will investigate the effects of donepezil on LPS-induced neuroinflammation via α7nAChR activation in a future study.

We and others have found that donepezil alters LPS-mediated proinflammatory cytokine levels and inflammation in vitro. Another study demonstrated that donepezil decreases LPS-induced Iba-1 immunoreactivity in wild-type mice [28]. In a 1-methyl-4-phenylpyridinium-induced mouse model of PD, donepezil treatment modulates proinflammatory cytokine levels (IL-6, IL-1β, and TNF-α) via inhibition of M1 polarization [49]. However, whether donepezil modulates LPS-induced microglial cell morphology and migration has not been studied in-depth. Consistent with previous findings, LPS-evoked Iba-1 immunofluorescence was reduced in donepezil-treated wild-type mice in the present study (Figure 5). Moreover, in wild-type mice, donepezil treatment significantly reduced LPS-stimulated Iba-1 hypertrophy and migration (Figure 5). The effects of donepezil on LPS-stimulated astrocyte activation in wild-type mice were not investigated in the present study because the effects of donepezil on primary astrocytes were smaller than those on microglia. Longer durations of treatment may be necessary to observe an effect of donepezil on LPS-mediated astrocyte activation and hypertrophy, and this possibility will be addressed in a future study. Overall, our findings and those of others suggest that donepezil regulates LPS-induced microglial neuroinflammation in wild-type mice.

COX-2 is a typical proinflammatory cytokine marker and may participate in memory and anxiety in addition to neuroinflammation [50]. Activated microglia release COX-2 in the early stages of neuroinflammation to mediate neuroinflammation and memory impairment [51]. In addition, the multifunctional cytokine IL-6 continuously activates the STAT signaling pathway to promote neuroinflammation [52]. Interestingly, in peripheral blood mononuclear cells from AD patients, donepezil decreases the induction of IL-1β and IL-6 expression [53]. This study is the first to investigate the effects of donepezil on LPS-stimulated proinflammatory cytokine levels in the brain. In wild-type mice, donepezil significantly downregulated LPS-induced COX-2 and IL-6 levels in the brain (Figure 6 and Figure 7). It is possible that donepezil affects other LPS-mediated proinflammatory cytokine levels (e.g., IL-1β, iNOS, and/or TNF-α) and NLRP inflammasome formation to modulate LPS-stimulated neuroinflammatory responses, which will be investigated in future studies.

Aβ plaques are a major hallmark of AD, and evidence suggests that Aβ-induced neuroinflammation plays a significant role in the pathophysiology of AD [54]. Neuroinflammatory reactions in glial cells and Aβ accumulation result in Aβ plaque formation, and repetition of this vicious cycle leads to neurodegenerative disease [55]. Consequently, regulation of neuroinflammatory responses is an important therapeutic strategy in AD. In 5xFAD mice, donepezil effectively downregulated Aβ-induced microglial and astrocyte activation (Figure 8 and Figure 9). Donepezil was previously shown to significantly inhibit Aβ-stimulated microgliosis in APP/PS1 mice by altering TNF-α and IL-1β proinflammatory cytokine levels [56]. Our results in a mouse model of AD further demonstrate that donepezil inhibits not only microgliosis but also astrogliosis. How does donepezil alter Aβ-stimulated astrogliosis in 5xFAD mice? Interestingly, in AD patients, donepezil alters astrocyte-induced neuroinflammatory responses by regulating kallikrein kinin signaling, a critical pathophysiological mediator of Aβ-induced neuroinflammation pathology [57]. In addition, donepezil modulates bradykinin-induced neuroinflammatory responses through nAChR and PI3K/AKT signaling in primary cortical astrocyte cells from Wistar rats [58]. These observations imply that donepezil may regulate Aβ-stimulated astrogliosis via nAChR and PI3K/AKT signaling and/or kallikrein kinin signaling in this mouse model of AD; the molecular mechanism of action will be investigated in a future study. Overall, our findings support anti-neuroinflammatory effects of donepezil in LPS-injected wild-type mice and a mouse model of AD.

The novelty of this study is that it explores the underlying mechanism of the effects of donepezil on the response to LPS in vitro and in vivo. First, we found that donepezil downregulates the LPS-induced neuroinflammatory response by inhibiting AKT/MAP kinase signaling and the NF-kB/STAT3-linked NLRP3 inflammasome. Donepezil was previously shown to modulate LPS-induced NF-kB signaling in the nucleus in vitro [3]. We further demonstrated that donepezil also affects LPS-mediated STAT3^ser727^ signaling to regulate neuroinflammatory responses. Second, we found differential effects of co-treatment with donepezil and rivastigmine compared with either AChE inhibitor alone. At doses up to 50 μM, the effects of rivastigmine on LPS-induced neuroinflammatory responses in BV2 microglial cells were smaller than those of donepezil. However, higher doses of rivastigmine (100 and 200 μM) significantly reduced LPS-induced increases in proinflammatory cytokine mRNA levels in BV2 microglial cells. Moreover, co-treatment with donepezil (50 μM) and rivastigmine (from 1–50 μM) did not have synergistic effects on LPS-mediated neuroinflammation in vitro. However, we speculate that co-treatment with higher doses of rivastigmine (100, 200 μM) and donepezil (10, 50 μM) may reduce neuroinflammation more effectively, which we will investigate in a future study. Third, we are the first to demonstrate that donepezil modulates LPS-evoked mRNA and protein levels of the proinflammatory cytokine COX-2 and IL-6 in wild-type mice. Finally, our study is the first to assess the effects of donepezil on Aβ-mediated neuroinflammation in 5xFAD mice, a mouse model of AD. Daily injection of donepezil for 14 consecutive days significantly diminished the Aβ-induced changes in microglial/astrocytic activation and morphology. Taken together, our results from two neuroinflammation-associated disease models (i.e., LPS, 5xFAD) support novel inhibitory effects of donepezil on LPS- and Aβ-induced neuroinflammatory responses.

## 4. Materials and Methods

### 4.1. Drugs

Donepezil (Cat no: 120014-70-3) and rivastigmine (Cat no:129101-54-8) were obtained from Neuland Laboratories Limited (Telangana, India) and Shodhana Laboratories Limited (Telangana, India), respectively. For in vitro assays, donepezil was dissolved in dimethyl sulfoxide (DMSO), and rivastigmine was dissolved in PBS. For in vivo assays, donepezil was dissolved in PBS and injected subcutaneously (1 mg/kg, s.c.) or intraperitoneally (1 mg/kg, i.p.) in wild-type and 5xFAD mice.

### 4.2. BV2 Cell Culture

BV2 microglial cells (generously gifted by Dr. Kyung-Ho Suk) were cultured in high-glucose Dulbecco’s Modified Eagle Medium (DMEM, Invitrogen, Carlsbad, CA, USA) with 5% fetal bovine serum (FBS, Invitrogen) and 1% penicillin G (100 U/mL) and streptomycin (100 mg/L) at 37 °C in a humidified environment with 5% CO_2_.

### 4.3. Mouse Primary Astrocyte Cell Culture

Primary astrocyte cells were isolated from postnatal day 1 C57/BL6 mice as previously described [22]. Whole mouse brains were minced through 70 μm nylon mesh and cultured in low-glucose DMEM with 10% FBS, 100 U/mL penicillin, and 100 μg/mL streptomycin in a 5% CO_2_ incubator. On the 14th day of growth, primary microglial cells were removed by shaking the mixed glial cells at 200 rpm on a rotary shaker at room temperature for 12 h. Primary astrocytes were subsequently detached by trypsin-EDTA, centrifuged at 2000 rpm for 30 min, and used for q-PCR.

### 4.4. MTT Assay

The cytotoxicity of donepezil and rivastigmine in BV2 microglial and primary astrocyte cells was evaluated using the reagent 3-(4,5-dimethylthiazol-2-yl)-2,5-diphenyltetrazolium bromide (MTT). BV2 microglial cells were seeded at 1 × 10^4^ cells/well in 96-well plates, incubated in serum-free medium for 1 h, and treated with donepezil or rivastigmine (1, 5, 10 and 50 μM) or the corresponding vehicle (1% DMSO or PBS, respectively) for 24 h. The MTT solution was then added and incubated for 2 h. After dissolving the formazan product crystals in DMSO for 20 min at room temperature, the absorbance at 570 nm (reference wavelength 660 nm) was measured using a SPECTROstar Nano microplate reader (BMG Labtech, Ortenberg, Germany).

### 4.5. Reverse transcription PCR (RT-PCR)

The mRNA levels of proinflammatory cytokines (IL-6, IL-1β, COX-2, and iNOS) in BV2 microglial cells treated with donepezil or rivastigmine were evaluated by RT-PCR. Briefly, the cells (2.5 × 10^5^ cells/mL in 12-well plates) were starved in serum-free media for 1 h before treatment with 200 ng/mL LPS or PBS for 30 min. The cells were then treated with drugs (donepezil, rivastigmine or donepezil + rivastigmine) or vehicle (1% DMSO) for 5.5 h. Total RNA extracted using TRIzol (Invitrogen, Carsbad, CA, USA) was used to synthesize cDNA via reverse transcription with the Superscript cDNA Premix Kit II (GeNetBio, Daejeon, Korea). PCR amplification was performed using 2xPrime Taq Premix (GeNetBio, Daejeon, Korea). The sequence information of the primers is shown in Table 1.

### 4.6. Real-Time Polymerase Chain Reaction (Quantitative PCR, q-PCR)

cDNA was synthesized with the Superscript cDNA Premix Kit II (GeNetBio, Daejeon, Korea) and analyzed by q-PCR using Fast SYBR Green Master Mix (Thermo Fisher Scientific, CA, USA) and a real-time PCR system (QuantStudio™ 5, Thermo Fisher Scientific, Waltham, MA, USA). The sequence information of the primers for q-PCR is shown in Table 2. The data were normalized using the GAPDH cycle threshold (Ct) value, and the fold change in donepezil-treated cells was calculated relative to the vehicle-treated control.

### 4.7. Intracellular ROS Content

A DCF-DA assay was used to evaluate the effects of donepezil or rivastigmine on intracellular ROS levels. In brief, BV2 microglial cells seeded in black 96-well plates at 2.0 × 10^4^ cells/well were treated with donepezil, rivastigmine, donepezil + rivastigmine or vehicle (0.5% DMSO) for 30 min before exposure for 23.5 h to 1 μg/mL LPS or PBS. DCF-DA (Invitrogen, Waltham, MA, USA) (10 μM) was then added and reacted for 40 min. The dichlorofluorescein (DCF) product was quantified by using a fluorometer (FlexStation 3 Multi-mode Microplate Reader, Molecular Devices, Jan Jose, CA, USA) with excitation at 485 nm and emission at 535 nm.

### 4.8. Mitochondrial Membrane Potential (MMP, ΔΨm)

To evaluate the effects of donepezil and/or rivastigmine on mitochondrial membrane potential, the dye JC-1 (Invitrogen, Waltham, MA, USA) was used as a lipophilic cationic probe. BV2 microglial cells were treated for 30 min with donepezil, rivastigmine, donepezil+rivastigmine or vehicle (0.5% DMSO) before exposure for 23.5 h to 1 μg/mL LPS or PBS. JC-1 solution (1 μM) was then added to each well and incubated for 10 min. Fluorescence emission was measured using a fluorometer (FlexStation 3 Multi-mode Microplate Reader, Molecular Devices, San Jose, CA, USA) with excitation at 485 nm and emission at 530 and 590 nm, and the 590 nm (orange-red)/530 nm (green) fluorescence ratio was calculated.

### 4.9. Immunocytochemistry (ICC) Assay

For ICC assays, BV2 microglial cells were exposed to 200 ng/mL LPS or PBS for 30 min before treatment with 50 μM donepezil or vehicle (1% DMSO) for 5.5 h. The cells were then fixed for 10 min in 4% PFA, washed with PBS, and incubated overnight at 4 °C with anti-CD11b (Abcam, Cambridge, UK) and anti-p-NFκBser536 (Cell Signaling Technology, Danvers, MA, USA) or anti-CD11b and anti-p-STAT3ser727 (Abcam, Cambridge, UK). After washing 3× for 10 min with PBS, the cells were incubated at room temperature with Alexa Fluor 488-conjugated anti-rat and Alexa Fluor 555-conjugated anti-rabbit antibodies (1:200, Molecular Probes, Eugene, OR, USA) for 2 h. The immunostained cells were washed with PBS for 10 min and mounted on glass slides using fluorescence mounting medium (Agilent Technologies, Santa Clara, CA USA). Images of the cells were acquired by fluorescence microscopy (DMi8, Leica Microsystems, Wetzlar, Germany), and fluorescence intensity was analyzed using Image J (Version 1.53a, National Institutes of Health, Bethesda, MD, USA).

### 4.10. Animals

All animal experimental protocols and guidelines were approved by the Korea Brain Research Institute Animal Care and Use committee (IACUC-21-00021). Wild-type mice and 5xFAD mice were housed in a pathogen-free facility at 22 ± 2 °C, 50 ± 5% humidity, and 12 h light/dark cycle with free access to chow diet and water.

### 4.11. LPS-Induced Neuroinflammation Mouse Model

Wild-type C57BL6/J mice (male, 8 weeks old) were purchased from Orient-Bio Company (Gyeonggi-do, Korea) and randomly allocated to 3 groups (control, LPS, and LPS + donepezil). The mice were injected with donepezil (1 mpk, s.c.) or vehicle (PBS) daily for 3 days. Thirty minutes after the last injection (day 3), LPS (10 mg/kg, i.p.) or PBS was injected. After 8 h, the mice were perfused with PBS and fixed with 4% paraformaldehyde (PFA) for immunohistochemistry (IHC) analysis. For post-fixation, brain tissue stored in 4% PFA for 24 h at 4 °C was immersed in 30% sucrose in PBS for 72 h. The tissues were then embedded in optimal cutting temperature compound and stored at −80 °C until frozen sectioning on a cryostat microtome.

### 4.12. Aβ-Induced Neuroinflammation Mouse Model (5xFAD Mice)

As a model of AD, 5xFAD APP/PS1 transgenic mice (Stock No. 34848-JAX, Jackson Laboratory, Bar Harbor, ME, USA) overexpressing human APP and PS1 with five familial AD mutations (B6.Cg-Tg (APPSwFlLon, PSEN1*M146L*L286V)6799Vas/Mmjax) were used. Genomic DNA extracted from the tail was used for mouse genotyping. To confirm the effect of donepezil on Aβ-induced neuroinflammation, 5xFAD mice (male, 3 months old) were injected daily with donepezil (1 mpk, i.p.) or vehicle for 2 weeks. Brain tissues from sacrificed mice were used in IHC with anti-Iba-1 and anti-GFAP.

### 4.13. Western Blotting and Nuclear Fractionation

The effects of donepezil on LPS-stimulated AKT/ERK signaling were confirmed by Western blotting. BV2 cells were lysed using lysis buffer (ProPrep, iNtRON Biotechnology, Inc., Seongnam, Korea) and centrifuged at 12,000 rpm for 15 min, and the supernatant was collected. The protein concentration was quantified in reference to a standard solution of BSA, and 20 μg of protein sample was separated by electrophoresis on an 8% SDS gel and transferred to a polyvinylidene difluoride (PVDF) membrane. After blocking with 5% skim milk or 5% BSA at room temperature for 1 h, the membrane was incubated overnight with anti-p-ERK (1:1000, Cell Signaling), anti-p-AKT^ser473^ (1:1000, Cell Signaling, Danvers, MA, USA), or anti-β-actin (l:1000, Santa Cruz, Dallas, TX, USA) at 4 °C. The next day, the membrane was incubated with HRP-conjugated goat anti-mouse IgG (1:1000, Enzo Life Sciences, Farmingdale, NY, USA) or HRP-conjugated goat anti-rabbit IgG (1: 1000, Enzo Life Sciences, Farmingdale, NY, USA) for 1 h, and detection was realized with ECL Western Blotting Detection Reagent (GE Healthcare, Chicago, IL, USA). The membranes were subsequently stripped and incubated with anti-ERK (1:1000, Cell Signaling) or anti-AKT (1:1000, Cell Signaling, Danvers, MA, USA) and developed accordingly. Images were acquired and analyzed using Fusion Capt Advance software (Vilber, Collegien, France).

### 4.14. Nuclear Fractionation

BV2 cells were lysed for 5 min in cytosol fractionation buffer (10 mM HEPES pH 8.0, 1.5 mM MgCl_2_, 10 mM KCl, 0.5 mM DTT, 300 mM sucrose, 0.5 mM PMSF and 0.1% NP-40) and centrifuged for 1 min at 10,000 rpm and 4 °C. The supernatant (cytosolic fraction) was set aside, and the nuclear fraction was obtained by lysing the pellet on ice for 15 min in nuclear fractionation buffer (10 mM HEPES pH 8.0, 100 mM KCl, 100 mM NaCl, 0.2 mM EDTA, 0.5 mM DTT, 0.5 mM PMSF and 20% glycerol) and centrifuging for 15 min at 10,000 rpm and 4 °C. Only the nuclear fraction was used in Western blotting to detect transcription factors.

### 4.15. Immunohistochemistry (IHC) Staining

Fixed brain tissues were sliced (30 μm) with a cryostat microtome (Leica CM1850, Wetzlar, Germany). The sections were blocked with 5% normal goat serum (Vector Laboratories, Burlingame, CA, USA) for 2 h at room temperature followed by immunostaining with primary antibodies (anti-Iba-1, anti-GFAP, anti-COX-2, or anti-IL-6) at 4 °C overnight. After washing 3× with PBST buffer, the tissues were incubated with fluorochrome (Alexa 555 or Alexa 488)-conjugated secondary antibodies for 2 h at room temperature. The immunostained tissue was mounted on glass slides using Vectashield mounting solution with DAPI (Vector Labs, Burlingame, CA, USA), and fluorescence microscopy images (DMi8, Leica Microsystems, Wetzlar, Germany) were analyzed using ImageJ (US National Institutes of Health, Bethesda, MD, USA). Table 3 provides information on the primary and secondary antibodies.

### 4.16. Statistical Analysis

Results are presented as means ± SED (* *p* < 0.05, ** *p* < 0.01, and *** *p* < 0.001), and the statistical significance of differences between groups was analyzed using Prism 7 (GraphPad Soft-ware version 7, San Diego, CA, USA). Unpaired two-tailed *t*-tests with Welch’s correction were used to compare two groups; to compare more than two groups, one-way analysis of variance (ANOVA) with Tukey’s or Dunnett’s multiple comparisons test was performed.

## 5. Conclusions

In BV2 microglial cells, the AChE inhibitor donepezil reduced LPS-stimulated proinflammatory cytokine levels by inhibiting NLRP3 inflammasome formation and modulating AKT/MAPK and NF-κB/STAT3 signaling. In addition, donepezil effectively attenuated LPS-induced oxidative stress by inhibiting intracellular ROS production and regulating mitochondrial membrane potential damage in vitro. In vivo, donepezil treatment significantly ameliorated LPS-stimulated microgliosis and COX-2 and IL-6 proinflammatory cytokine levels in the brain in wild-type mice and Aβ-stimulated microgliosis and astrogliosis in 5xFAD mice. In summary, donepezil modulates LPS- and Aβ-induced neuroinflammatory responses in the brain in wild-type mice and a mouse model of AD, indicating that donepezil may be a useful drug for neuroinflammation-associated diseases.

## Figures and Tables

**Table 1:** Primer sequences for RT-PCR analysis of proinflammatory cytokines.

Gene		Primer Sequence (5′–3′)
COX2	Forward	GCC AGC AAA GCC TAG AGC AA
	Reverse	GCC TTC TGC AGT CCA GGT TC
IL-6	Forward	GCC AGC AAA GCC TAG AGC AA
	Reverse	GCC TTC TGC AGT CCA GGT TC
IL-1β	Forward	AGC TGG AGA GTG TGG ATC CC
	Reverse	CCT GTC TTG GCC GAG GAC TA
iNOS	Forward	CCG GCA AAC CCA AGG TCT AC
	Reverse	GCA TTT CGC TGT CTC CCC AA
GAPDH	Forward	CAG GAG CGA GAC CCC ACT AA
	Reverse	ATC ACG CCA CAG CTT TCC AG

**Table 2:** Primer sequences of primers used for q-PCR.

Gene		Primer Sequence (5′–3′)
GAPDH	Forward	TGG GCT ACA CTG AGG ACC ACT
	Reverse	GGG AGT GTC TGT TGA AGT CG
iNOS	Forward	GGA TCT TCC CAG GCA ACC A
	Reverse	TCC ACA ACT CGC TCC AAG ATT
COX-2	Forward	CCA CTT CAA GGG AGT CTG GA
	Reverse	AGT CAT CTG CTA CGG GAG GA
IL-6	Forward	CCA CGG CCT TCC CTA CTT C
	Reverse	TTG GGA GTG GTA TCC TCT GTG A
IL-1β	Forward	TTG ACG GAC CCC AAA AGA TG
	Reverse	AGG ACA GCC CAG GTC AAA G
Pro-IL-1β	Forward	TCT TTG AAG TTG ACG GAC CC
	Reverse	TGA GTG ATA CTG CCT GCC TG
NLRP3	Forward	TCC ACA ATT CTG ACC CAC AA
	Reverse	ACC TCA CAG AGG GTC ACC AC

**Table 3:** Primary and secondary antibodies used in IHC analysis.

**Primary Antibodies**	**Host Species**	**Dilution**	**Manufacturer**	**Catalog No.**
Anti-Iba-1	Rabbit	1:500	Wako	019-19741
Anti-GFAP	Rabbit	1:500	Neuromics	RA22101
Anti-IL-6	Mouse	1:50	Santa Cruz	SC-57315
Anti-COX-2	Rabbit	1:200	Abcam	ab15191
**Secondary Antibodies**		**Dilution**	**Manufacturer**	**Catalog No.**
Goat anti-rabbit IgG, 555		1:200	Invitrogen	A21428
Goat anti-rabbit IgG, 488		1:200	Invitrogen	A11008
Goat anti-mouse IgG, 488		1:200	Invitrogen	A11001

## Data Availability

All data generated and/or analyzed during this study are included in this published article.
